# Wearable-Sensor-Based Analysis of Aerial Archimedean Spirals for Early Detection of Parkinson’s Disease

**DOI:** 10.3390/s25247579

**Published:** 2025-12-13

**Authors:** Hao Shi, Sanyun Chen, Zhuoying Jiang, Yuting Wang

**Affiliations:** 1 School of Information Engineering, Hangzhou Medical College, Hangzhou 311399, China; chensanyun@hmc.edu.cn (S.C.); wangyuting@hmc.edu.cn (Y.W.); 2 School of Public Health, Hangzhou Medical College, Hangzhou 311399, China; jiangzhuoying@hmc.edu.cn

**Keywords:** Parkinson’s disease, IMU, early detection, attention mechanism, LAFNet, Archimedean spiral, Kalman filter

## Abstract

Parkinson’s disease (PD) is a progressive neurodegenerative disorder whose early symptoms, especially mild tremor, are often clinically imperceptible. Early detection is crucial for initiating neuroprotective interventions to slow dopaminergic neuronal degeneration. Current PD diagnosis relies predominantly on subjective clinical assessments due to the absence of definitive biomarkers. This study proposes a novel approach for the early detection of PD through a custom-developed smart wristband equipped with an inertial measurement unit (IMU). Unlike previous paper-based or resting-tremor approaches, this study introduces a mid-air Archimedean spiral task combined with an attention-enhanced Long Short-Term Memory (LSTM) architecture, enabling substantially more sensitive detection of subtle early-stage Parkinsonian motor abnormalities. We propose LAFNet, a model based on an attention-enhanced LSTM network, which processes motion data that has been filtered using a Kalman algorithm for noise reduction, enabling rapid and accurate diagnosis. Clinical data evaluation demonstrated exceptional performance, with an accuracy of 99.02%. The proposed system shows significant potential for clinical translation as a non-invasive screening tool for early-stage Parkinson’s disease (PD).

## 1. Introduction

Parkinson’s disease (PD) ranks as the second most common neurodegenerative disorder globally. Recent 2023 data from the World Health Organization (WHO) indicate that the prevalence of PD has doubled in the past 25 years, with incidence expected to rise substantially over the next three decades [1]. Notably, disability and mortality attributable to PD are increasing at a faster rate than those of any other neurological condition, a trend driven mainly by delayed diagnosis, frequent misdiagnosis, and inadequate treatment coverage [2]. The disease progression entails a gradual loss of motor function, accompanied by muscle atrophy and joint rigidity, often culminating in permanent bedridden states. PD is characterized by a prolonged latency period, during which early symptoms are typically subtle and go unrecognized. By the time a diagnosis is formally established using standardized clinical rating scales, the condition has often advanced significantly, requiring higher dosages of dopamine agonists. This frequently leads to diminished therapeutic responses and the emergence of motor complications, which in turn accelerate disease progression in later stages [3]. The absence of a definitive biomarker means that diagnosis continues to rely on the identification of core motor symptoms [4]. In the lack of objective early screening tools, a majority of individuals are diagnosed only during middle or late stages, long after the optimal window for intervention has passed.

Although PD is the most representative disorder within the spectrum of dopaminer-gic degenerative disorders (DDD), it constitutes only one subtype of a broader group of neurodegenerative conditions with overlapping clinical manifestations. Several disorders—including essential tremor (ET), multiple system atrophy (MSA), progressive supranuclear palsy (PSP), and dementia with Lewy bodies (DLB)—may present with brady-kinesia, rigidity, or gait abnormalities similar to those observed in PD, especially during the prodromal and early symptomatic stages. Importantly, resting tremor, often regarded as a hallmark of PD, does not consistently appear in the earliest phases of the disease and may be completely absent in certain PD phenotypes [5]. Consequently, early differential diagnosis based solely on motor symptoms remains clinically challenging, leading to frequent misdiagnosis or delayed diagnosis in routine practice. Therefore, developing objective, quantitative biomarkers for distinguishing PD from other DDDs is of substantial clinical value.

Modern diagnostic approaches increasingly incorporate neuroimaging, particularly positron emission tomography (PET), which evaluates dopaminergic terminal integrity through radiotracer uptake. PET imaging is currently the only pathognomonic technique capable of visualizing nigrostriatal degeneration and confirming PD with high sensitivity. However, PET is costly, requires specialized equipment, involves ionizing radiation, and is not widely available in primary or community healthcare settings. As a result, most patients are still diagnosed based on clinical criteria supported by auxiliary assessments—including endocrine markers, selected neurohormonal panels, and immunobiochemical indicators—none of which provide conclusive evidence on their own. In contrast, the method presented in this study offers a non-invasive, low-cost, and easily deployable solution capable of capturing subtle movement abnormalities that precede overt motor symptoms. By leveraging wearable sensors and data-driven modeling, our approach provides an accessible and objective diagnostic option that complements current neuroimaging-based strategies.

The main contributions of this work are as follows:

(1) Mid-air Archimedean Spiral Acquisition for Early PD Detection: Unlike prior studies that rely on paper-based spirals or resting tremor recordings, we propose a mid-air Archimedean spiral task captured by a custom-designed wrist-worn IMU device. Removing the physical drawing surface increases motor-load sensitivity, enabling the system to capture subtle prodromal tremor signatures that conventional paper tasks often fail to reveal.
(2) A Custom IMU Wristband for High-Fidelity Kinematic Sensing: We developed a lightweight, low-cost smart wristband integrating a tri-axial accelerometer and gyroscope. This device continuously streams high-resolution kinematic data during free-hand tracing, offering an objective and scalable alternative to clinical motor assessments.
(3) LAFNet: An Attention-Enhanced LSTM Model for Tremor Representation: We introduce LAFNet, a fused-attention LSTM architecture optimized for noisy physiological motion data. The linear attention module adaptively emphasizes discriminative temporal segments, allowing the model to extract disease-relevant micromovement patterns that are overlooked by traditional RNN/LSTM networks.
(4) Superior Diagnostic Accuracy Compared with Existing Approaches: Through extensive clinical evaluation, LAFNet achieved an accuracy of 99.02%, outperforming four model variants and exceeding the performance of state-of-the-art tremor-based PD detection methods. The model also significantly surpasses paper-based spiral tasks (accuracy: 88.30%), demonstrating the effectiveness and uniqueness of the proposed mid-air acquisition paradigm.

## 2. Related Work

### 2.1. Diagnosis of PD

PD is a progressive neurodegenerative disorder that develops over decades. Its early symptoms advance gradually but cumulatively lead to escalating disability, posing a growing socioeconomic burden [6]. Despite its significant impact, no validated biomarker or definitive laboratory test exists to confirm the diagnosis. Currently, PD identification remains reliant on clinical assessment by neurologists, who integrate medical history, reported symptoms, and findings from neurological examinations to formulate a diagnosis.

Current approaches for tremor frequency analysis in PD diagnosis include gait analysis [7], hand-drawn-pattern evaluation [8,9], and wrist tremor monitoring. For example, reference [10] proposed the use of sensors to capture gait parameters of patients, including walking speed, stride length, and cadence. Similarly, Hammoud et al. recorded wrist-motion signals from patients and controls, benchmarking multiple binary classifiers on the resulting feature set [11]. Other studies have leveraged wearable sensors combined with machine learning models for quantitative tremor assessment [12]. Motion-capture systems have also been introduced to automate the rating of bradykinesia and finger-tapping tasks [13]. Further advancing this domain, features derived from higher-order kinematics during Archimedean spiral drawing have been proposed to enhance diagnostic accuracy [14]. Complementary approaches include the use of portable electromyography (EMG) units to differentiate PD resting tremor from essential tremor [15] and the development of tremor percentage metrics derived from accelerometer data for continuous monitoring [16]. More recently, deep learning techniques such as convolutional neural networks (CNN) have been applied to classify distinct tremor types in PD, including resting, postural, and action tremors [16].

Tracing Archimedean spirals has recently gained traction as a non-invasive diagnostic aid. Saha et al. fused spiral, meander, and micrographia tasks within a multimodal framework, improving detection accuracy [17]. Gupta et al. extracted novel image-based features from spiral traces to strengthen diagnostic reliability [18]. Jenei et al. fused multiple spiral-drawing modalities to improve recognition efficiency further [19]. In 2024, Saetee et al. optimized PD diagnostic accuracy through digital Archimedean curve handwriting analysis [20]. Collectively, these studies underscore the growing promise of algorithm-assisted Archimedean-spiral analysis for early PD detection.

### 2.2. Long Short-Term Memory Neural Networks

LSTM networks have demonstrated significant potential in analyzing tremor data for diagnosing PD and essential tremor (ET). For example, Oktay et al. applied a convolutional LSTM to distinguish PD from ET using wrist-worn accelerometer signals [21]. Similarly, Otero et al. enhanced ET diagnostic accuracy by integrating empirical mode decomposition (EMD)-based data augmentation with LSTM analysis of handwriting samples [22]. Ibrahim et al. and Farhani et al. utilized stacked bidirectional LSTM architectures for real-time classification of PD tremor episodes [23,24]. Further expanding these applications, Hathaliya et al. incorporated blockchain technology with deep learning to improve tremor classification performance [25]. Collectively, these studies underscore the efficacy and robustness of LSTM networks in processing tremor signals, offering a promising foundation for precision diagnostics in movement disorders.

LSTM is a recurrent neural network variant specifically designed to capture both short-term and long-term temporal dependencies [26,27,28]. Whereas vanilla RNNs suffer from exponential gradient decay, LSTM introduces a gating subsystem that regulates information flow. At each time step, the input, forget, and output gates determine which values to retain, update, or discard, shielding the gradient from irrelevant detail. Unlike standard RNN cells, which overwrite their hidden state, LSTM maintains a separate cell state that can propagate unchanged over arbitrary intervals, allowing earlier features to influence distant time steps. This selective reuse enables the network to balance historical and instantaneous evidence, a property critical for sequences with long and irregular latent structure.

LSTM-based sequence models have proven effective for capturing long-range temporal dependencies across diverse vision tasks. In video restoration and deraining, Enhanced Spatio-Temporal Interaction Networks (ESTINet) [29] couple convolutional LSTM with residual backbones to learn powerful spatio-temporal features while maintaining efficiency, illustrating how recurrent units can exploit inter-frame correlations for robust sequence enhancement. In facial expression analysis, part-based hierarchical bidirectional RNNs (PHRNN) [30] demonstrate that structured recurrent designs over semantically meaningful parts provide strong temporal representations complementary to CNN appearance cues. More broadly, surveys of deep image/video deblurring document the growing role of recurrent layers within restoration architectures (e.g., scale-recurrent networks and RNN variants) [31], underscoring their utility for modeling dynamics in sequential data.

### 2.3. Kalman Filter

The Kalman filter serves as a critical technical component in transforming raw, noisy sensor data into high-quality digital biomarkers suitable for clinical analysis. It directly determines the accuracy of your early Parkinson’s disease detection. The Kalman filter is a recursive estimator that operates through a cyclic two-step process of prediction and update [32,33,34]. At every moment, it treats the system state as a Gaussian variable, propagating its mean and covariance through the dynamics and then updating both with the latest measurement likelihood. By fusing data across time in this way, exploiting joint rather than isolated distributions, it yields estimates whose mean squared error is provably smaller than that of any single snapshot inference, turning incomplete and noisy sequences into optimally filtered trajectories. The Kalman filter algorithm iterates between two distinct stages: prediction and update. During the prediction phase, the previous state estimate and its associated error covariance are propagated forward using the system’s dynamic model, yielding a prior state estimate and a predicted error covariance [35]. In the subsequent update phase, the Kalman gain is computed to balance the previous estimate with the incoming measurement. This gain is applied to refine the state estimate and update the error covariance, resulting in a posterior estimate that is fed into the next iteration [36].

## 3. Methods

This paper proposes an early-stage PD detector centred on an LSTM backbone enhanced by a fused attention mechanism. Five variants were trained and compared: LAFNet, which augments LSTM with linear attention; TAFNet, a stacked LSTM whose attention weights pass through a Tanh gate; LDLFNet, a stacked LSTM regularised by dropout; LDLAFNet, which unites linear attention with dropout; and LDTAFNet, where Tanh-gated attention and dropout are combined. LAFNet outperformed the rest, achieving 99.02% accuracy.

The following section discusses the rationale for diagnosing PD based on Archimedes spirals drawn in mid-air and the data preprocessing steps. Subsequently, the LAFNet pipeline is examined layer by layer: Input, LSTM, attention, dropout, and fully connected layers.

### 3.1. Early Detection of PD by Assessing Air-Drawing Archimedean Spiral with the Finger

Bradykinesia, a core motor manifestation and mandatory diagnostic criterion for PD, serves as a primary indicator of Parkinsonian syndrome—defined by its presence in combination with either resting tremor or rigidity [4]. The objective quantification of movement slowing thus constitutes a fundamental component of clinical assessment. As an essential diagnostic feature, bradykinesia is systematically evaluated in research studies, progression scales, and routine clinical monitoring, providing a basis for detection, quantification, and longitudinal tracking of the disease.

### 3.2. Smart Bracelet and Data Acquisition

**Figure 1:**
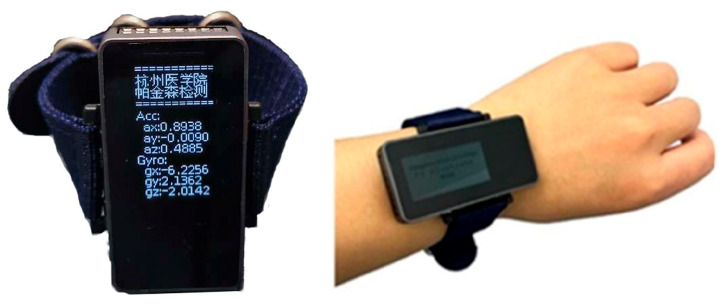
Smart data collection wristband.

This research utilizes a custom hardware module designed for collecting patient motion data. Built around an ESP32 microcontroller and an IMU sensor (MPU6050) as its core components, the system operates through I^2^C communication between the ESP32 and the MPU6050. This protocol enables acquisition of six-axis motion data via the sensor’s integrated triaxial accelerometer and gyroscope, providing linear acceleration (ax, ay, az) and angular velocity (gx, gy, gz). As shown in Figure 1, the device is a smart wristband designed primarily for high-fidelity motion data collection.

### 3.3. Data Preprocessing

**Figure 2:**
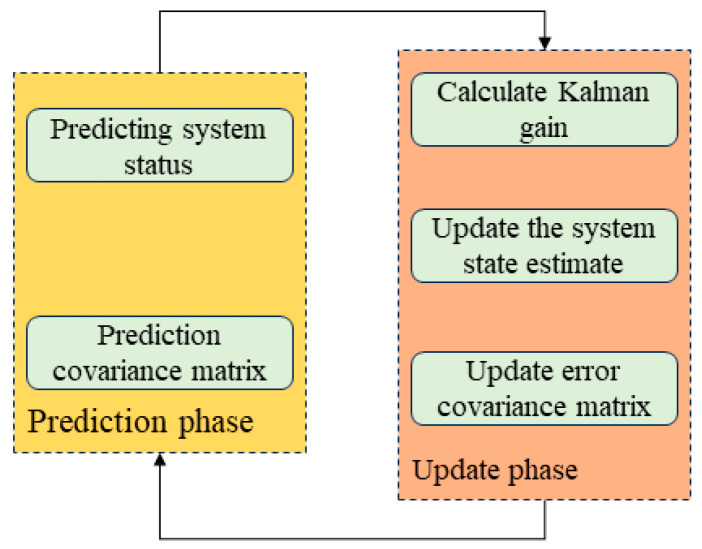
Kalman filter algorithm flowchart.

The raw gyroscope data are inherently contaminated by significant noise and suffer from drift that accumulates over time. When integrated, even minor noise can lead to substantial angular errors, resulting in a severely distorted reconstructed spiral trajectory that fails to accurately capture patient tremor and bradykinesia. The Kalman filter addresses this by optimally combining model predictions with sensor observations, effectively attenuating high-frequency random noise and mitigating drift. Thereby, it yields a cleaned, stabilized orientation or angular signal that closely approximates the true motion. The Kalman filter algorithm flowchart is illustrated in Figure 2.

The Kalman filter is naturally split into two steps: time update and measurement update. During the time update, the previous state estimate and its covariance are propagated forward to produce the prior for the current instant. The measurement update then fuses this prior with the newest observation: it forms the innovation by comparing the predicted and actual measurements, computes the Kalman gain that weights the relative trust in the model versus the data, and applies this gain to yield the posterior state estimate. The time update equations are given below.

(1) $${\hat{x}}_{\bar{k}}=A{\hat{x}}_{k-1}+Bu_{k-1}$$

(2) $$P_{\bar{k}}=AP_{k-1}A^{T}+Q$$

where ${\hat{x}}_{k-1}$ and ${\hat{x}}_{\bar{k}}$ represent the posterior state estimates at time k−1 and k, respectively, which are among the outputs of the Kalman filter. ${\hat{x}}_{\bar{k}}$ denotes the prior state estimate at time k, obtained as a result of the prediction step of the filtering process. $P_{k-1}$ and $P_{k}$ represent the posterior estimate covariances at time k−1 and k, corresponding to the covariances of ${\hat{x}}_{k-1}$ and ${\hat{x}}_{\bar{k}}$, respectively. These values indicate the uncertainty of the state estimates and are also among the filter’s outputs. Where B is the matrix that transforms the input into the state. The state update equations are given in (3)–(5).

(3) $$K_{k}=\frac{P_{\bar{k}}H^{T}}{HP_{\bar{k}}H^{T}+R}$$

(4) $${\hat{x}}_{k}={\hat{x}}_{\bar{k}}+K_{k}(z_{k}-H\;{\hat{x}}_{\bar{k}})$$

(5) $$P_{k}=(I-K_{k}H)P_{\bar{k}}$$

where *H* represents the state-to-observation matrix, which maps the state space to the measurement space and serves as one of the prerequisites for the filtering process. $z_{k}$ denotes the measurement value, acting as the input to the filter. $K_{k}$ is the Kalman gain matrix, an intermediate computation result of the filtering algorithm. R represents the measurement noise covariance. The term $z_{k}-H{\hat{x}}_{\bar{k}}$ corresponds to the residual between the actual observation and the predicted observation. This residual, combined with the Kalman gain, corrects the prior state estimate to obtain the posterior state estimate.

### 3.4. LAFNet Architecture

**Figure 3:**
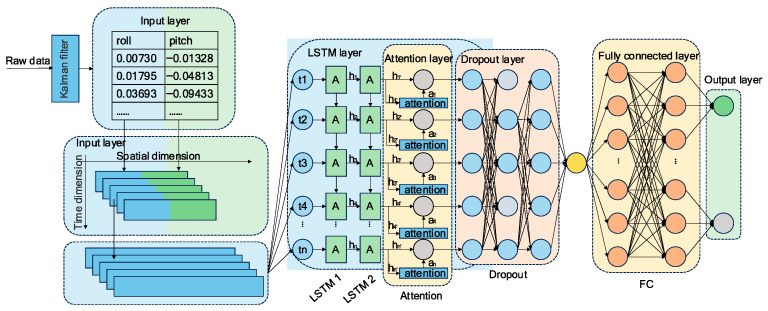
LSTM network model structure diagram.

This paper presents a novel model based on an attention-enhanced long short-term memory network for diagnosing early-stage PD. The proposed LAFNet model architecture, shown in Figure 3, consists of five core components: an input layer, an LSTM layer, an attention mechanism, a dropout layer, and a fully connected layer. Designed to process sequential data (such as IMU sensor readings, roll, and pitch), this architecture captures both long-term and short-term temporal dependencies while selectively focusing on the most informative time steps through the attention mechanism.

At the input layer, multivariate time series data are received as sequential feature vectors. Each sequence contains measurements spanning multiple time steps, preserving the inherent spatio-temporal structure of the sensor data.

The encoding stage utilizes multiple stacked LSTM layers to model temporal dependencies within the input sequences. This hierarchical architecture enables the network to capture evolving features across different time scales, significantly enhancing its ability to learn complex temporal patterns. After LSTM processing, an attention mechanism is applied to the output sequences. This component computes attention weights that quantify the relative importance of each time step, allowing the model to focus selectively on the most clinically relevant segments of the movement sequence.

To mitigate overfitting and enhance the model’s generalization ability, a Dropout layer is added after the attention mechanism. During training, this layer randomly discards a portion of neurons to prevent the model from becoming overly reliant on specific features. The output from the dropout layer is fed into a fully connected classification layer, which converts the learned representations into predicted scores for the target categories.

Traditional RNNs and LSTMs process every past hidden state with equal weight, a feature that buries faint but critical transients beneath redundant history. The added attention gate breaks this symmetry by reweighting each time step on the fly, amplifying microseconds that carry a disease signature while downgrading the rest. Stacking the LSTMs deepens the temporal code, and the attention layer distils it, so only the most informative frames reach the classifier. The result is an architecture tuned for noisy sensor or physiological streams, delivering predictions that are both more accurate and more explainable than those of its conventional counterparts.

In summary, the proposed architecture integrates the complementary strengths of deep recurrent networks and attention-based sequence modeling, yielding a high-performance framework that significantly enhances both predictive accuracy and model interpretability in time-series analysis.

#### 3.4.1. Input Layer

The model accepts multivariate time-series data as input, with each temporal instance comprising multiple feature dimensions corresponding to roll and pitch angular measurements. These values capture the spatial orientation and rotational dynamics of the movement. To maintain temporal coherence, the input is structured as a two-dimensional matrix, where rows represent sequential time steps and columns correspond to distinct orientation features. As illustrated in the left panel of Figure 3, the data are organized chronologically along the temporal axis.

#### 3.4.2. LSTM Layer

To capture dependency information within sequences, an LSTM network is employed as the feature extraction module. Thanks to its gated memory structure, LSTM can effectively capture long-term temporal dependencies and dynamic features in sequential data. Suppose the input sequence is denoted as $X=\{x_{1},x_{2},x_{3},\cdots ,x_{T}\}$, the LSTM computes a hidden state $h_{t}$ at each time step as follows.

(6) $$h_{t}=LSTM(x_{t},h_{t-1},c_{t-1})$$

where $h_{t}$ represents the current hidden state and $c_{t}$ denotes the memory cell state. The output of the entire LSTM layer is given by (7).

(7) $$H=\{h_{1},h_{2},\cdots ,h_{T}\},H\in {\mathbb{R}}^{T\times H_{d}}$$

where $H_{d}$ denotes the dimensionality of the hidden state.

#### 3.4.3. Attention Layer

To enhance the model’s capacity to focus on clinically relevant information, an attention mechanism is incorporated following the LSTM layer. We employ a linear attention approach that performs direct weighted aggregation of hidden states across time steps. This mechanism computes a weighted sum of the temporal sequences, effectively highlighting the most discriminative segments for PD detection. The attention weights are calculated according to (8).

(8) $$a_{t}=W_{a}h_{t}+b_{a},W_{a}\in {\mathbb{R}}^{1\times H_{d}},b_{a}\in \mathbb{R}$$

The attention weight $a_{t}$ is a scalar value representing the importance of the corresponding time step. After generating the attention weights, the weighted results from each time step are summed to form a context vector, as shown in (9). During model training, the parameters $W_{a}$ and $b_{a}$ are automatically learned to enable the final context vector to more effectively capture the global features of the entire sequence.

(9) $$context=\sum_{t=1}^{T}a_{t}\cdot h_{t}$$

#### 3.4.4. Dropout Layer

To mitigate overfitting during training, a dropout layer is applied after the attention module. This regularization technique works by randomly suppressing the outputs of a subset of neurons, thereby reducing the model’s tendency to rely excessively on specific features. The output of the dropout layer is expressed in (10), where $H_{d}$ represents the dimension of the context vector.

(10) $$y=Dropout(x),x\in {\mathbb{R}}^{H_{d}}$$

#### 3.4.5. Fully Connected Layer

The feature vector output by dropout is fed into a fully connected layer to perform the final classification task. This layer consists of a Dense layer followed by a softmax activation function, which outputs probability values. Assuming the context vector is denoted as x, the output is given by (11), where W and b represent the weight matrix and bias term, respectively, and σ denotes the softmax activation function. The final output is the predicted probability distribution.

(11) z=σ(Wx+b)

### 3.5. Attention Interpretability Analyses

To enhance clinical transparency, we computed the contributions of temporal segments and sensor channels to the PD/healthy classification.

(1) Temporal attention maps: From LAFNet’s final attention module we extracted frame-level weights $\alpha_{t}$. For each sequence, we normalized
  (12) $${\hat{\alpha }}_{t}=\frac{\alpha_{t}}{\sum_{t}\alpha_{t}}$$
  upsampled ${\hat{\alpha }}_{t}$ to the raw sampling rate by linear interpolation, and applied light smoothing (Savitzky–Golay, window 51, order 3). We overlaid ${\hat{\alpha }}_{t}$ as color intensity on roll/pitch traces (Angle, °) to highlight high-weight windows.
(2) Channel-wise importance. For each input axis (acc-x/y/z, gyro-x/y/z), we computed importance as the time average of normalized attention,
  (13) $$I_{c}=\frac{1}{T}\sum_{t}{\hat{\alpha }}_{t,c}$$
  normalized $I_{c}$ to percentages per sequence, then averaged across subjects to obtain group statistics.

## 4. Experiment and Result

To evaluate the proposed method, tremor data were collected from patients at Hangzhou Lin’an People’s Hospital. Ethical approval was granted by the Institutional Review Board (IRB) of Hangzhou Medical College (Ethical Approval No. LL2025-009). These data were utilized to train and evaluate the five model variants presented in this study.

We implemented the LAFNet models using the PyTorch 2.5.1 framework and trained them on a single NVIDIA RTX 4070 GPU (MSI Corporation, Suzhou, China). The experimental data were collected using smart wristbands from participants comprising two groups: healthy controls, patients with early-stage PD. The data were sampled at 330 Hz, with each recording lasting approximately 60 s, resulting in 19,800 data packets per sample. Each packet includes six motion parameters: ax, ay, az, gx, gy, and gz, corresponding to triaxial linear acceleration and angular velocity. We employed the Adam optimizer with a learning rate of 1 × 10^−3^, momentum of 0.9, and weight decay of 1 × 10^−5^. Unless otherwise specified, all models were trained for 300 epochs under consistent settings. The following sections detail the experimental setup and procedures.

### 4.1. Comparison of Paper-Based Spiral Drawings Between PD Patients and Healthy Controls

**Figure 4:**
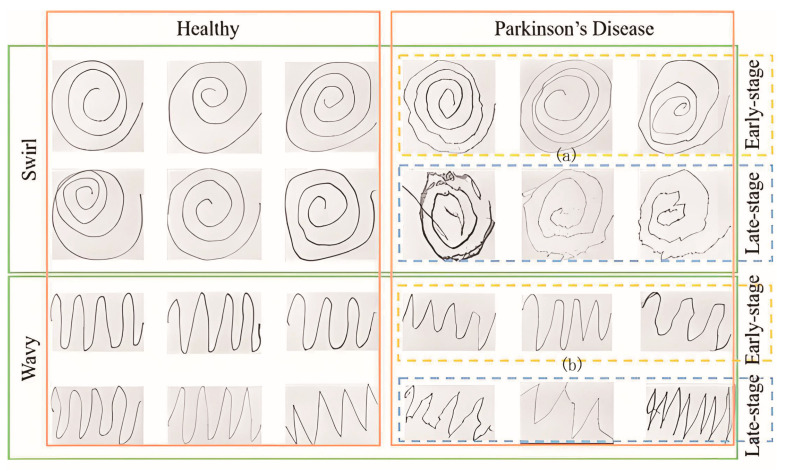
Comparison of hand-drawn swirls and wavy lines across different stages of PD.

Drawing tasks, such as tracing the Archimedean spiral, are widely employed in the early diagnosis and progression assessment of PD to capture subtle motor impairments [37], as shown in Figure 4.

Figure 4 illustrates that the spiral and wave patterns drawn by healthy individuals exhibit smooth and regular trajectories, reflecting well-coordinated motor function and precise movement control. In contrast, patients with PD demonstrate a progressive decline in drawing performance, characterized by clearly observable kinematic alterations.

In the early stages of PD, spiral patterns begin to exhibit slight distortions and irregularities in lines, indicating the initial deterioration of motor precision. Similarly, wavy patterns display fluctuations and inconsistencies in amplitude, suggesting the emergence of tremor or rigidity, although overall line continuity remains largely preserved. These changes reflect early motor impairment, likely associated with symptoms such as bradykinesia and mild stiffness. However, early Parkinsonian symptoms, including tremor and rigidity, are often subtle. In drawing tasks, these may manifest as minor line tremors or uneven spacing, which can be difficult to distinguish from the natural variability observed in healthy subjects due to individual drawing habits or temporary fatigue. For example, while drawings from healthy individuals generally appear smooth, some also show slight imperfections. Conversely, although drawings from right-handed PD patients often exhibit visible tremors, particular examples, such as Figure 4 marked a, b, show minimal differences from healthy traces, making accurate visual classification challenging without quantitative analysis.

Additionally, manual tracing provides no quantitative measurement of tremor frequency, inter-loop spacing, or smoothness of curvature. As a result, examiners must rely on subjective thresholds, leading to considerable inter-rater variability. Similarly, waveform analysis offers limited utility: the subtle amplitude fluctuations visible in the bottom-right plot are comparable in magnitude to physiological jitter observed in healthy controls, and therefore lack sufficient specificity to serve as a reliable diagnostic indicator. Consequently, conventional paper-based Archimedean spiral tests alone are inadequate for detecting incipient PD.

In later stages of PD, patients exhibit markedly impaired drawing performance. Spiral patterns display substantial irregularities and jagged trajectories, indicating a severe deterioration in movement precision. Similarly, wavy patterns become fragmented and inconsistent, reflecting pronounced motor deficits as patients experience considerable difficulty in controlling hand movements. Advanced motor impairments, including rigidity, tremor, and bradykinesia, collectively contribute to a pronounced degradation in graphical output, resulting in distorted and often incomplete traces.

Quantitative analysis of on-air drawing tasks provides a novel approach for early detection and long-term management of PD. Algorithms capable of detecting micrometric tremors, hesitations, and trace tapering can identify disease progression months before clinical manifestation of overt symptoms. These objective metrics also help predict which patients are likely to benefit from medication adjustments or physical therapy interventions. By replacing subjective visual assessment with standardized digital measurement, this method eliminates observer bias and generates reproducible data to support both accurate diagnosis and personalized treatment strategies.

### 4.2. Study Population and Data Collected

We enrolled 50 patients with idiopathic Parkinson’s disease (PD) and 51 healthy individuals as controls. All PD diagnoses were made by experienced neurologists according to current clinical diagnostic criteria, based on a detailed medical history, neurological examination, and exclusion of secondary or atypical parkinsonism. Patients with a history of stroke, other major neurological or psychiatric disorders, or severe systemic disease were excluded. Healthy controls were recruited from the community and hospital staff and were screened to exclude any history of movement disorders or major neurological disease.

Disease severity in the PD group was graded using the Hoehn and Yahr (H&Y) scale, a global staging system ranging from stage 1 (unilateral involvement only) to stage 5 (wheelchair-bound or bedridden). In this study, only patients in H&Y stages 1–2 were included, corresponding to early-stage PD with unilateral or mild bilateral motor signs and without postural instability. We note that the H&Y scale is not universally applicable to all PD phenotypes and provides only a coarse index of global severity; therefore, we additionally collected detailed clinical information to better characterize our cohort.

For each PD patient, structured anamnesis data were obtained, including disease duration, side and type of initial motor symptoms, family history of PD, comorbidities, and current dopaminergic medication. The final diagnostic procedure (clinical criteria used, treating neurologist’s judgement, and, when available, documented response to dopaminergic therapy) was recorded in the medical chart. Executive function at the time of examination was clinically judged to be sufficient to perform the drawing tasks, and patients with obvious executive or cognitive impairment that could interfere with task performance were not included. The demographic and clinical characteristics of all participants are summarized in Table 1.

### 4.3. Data Normalization

Before model training, the collected data underwent comprehensive analysis and preprocessing to enhance the accuracy and generalization capability of the detection algorithm. Raw data acquired from the sensing device were stored in text files containing sequentially arranged values for ax, ay, az, gx, gy, and gz. These data were processed through a structured pipeline beginning with a cleaning phase, where custom functions identified and removed abnormal or invalid samples. This process yielded a curated dataset of validated raw motion data suitable for subsequent feature extraction and model training.

The input data underwent normalization to enhance training efficiency and improve model performance. This preprocessing step maps the data to a standardized numerical range, typically [0, 1], mitigating the influence of varying dimensional scales and value ranges. Such normalization promotes improved model convergence, training stability, and computational efficiency. The Min-Max normalization method was employed for this purpose as follows.

(14) $$x^{\prime }=\frac{x-x_{\min}}{x_{\max}-x_{\min}}$$

where $x_{min}$ and $x_{max}$ represent the minimum and maximum values of a feature in the entire dataset x, respectively, while $x^{\prime }$ denotes the normalized value. Since the data consists of motor sensor signals from PD patients, the amplitude of such signals holds explicit meaning, representing actual transformations in movement intensity. Therefore, the normalization process requires particular attention to whether it affects the proportional relationships of the original data’s amplitudes. Compared to methods like Z-score normalization, Min-Max normalization offers a significant advantage in preserving the original proportional relationships of the signals. This ensures that the critical information carried by the biological signals at the physical level is retained.

### 4.4. Kalman Filter and Analysis

Following data cleaning, attitude estimation was performed on the curated dataset. A Kalman filter was applied to process the six-axis sensor data, converting the raw measurements into three Euler angles: roll, pitch, and yaw, representing rotations around the Z-axis, X-axis, and Y-axis, respectively. From these derived angles, roll and pitch values were selected as the primary parameters for subsequent analysis.

**Figure 5:**
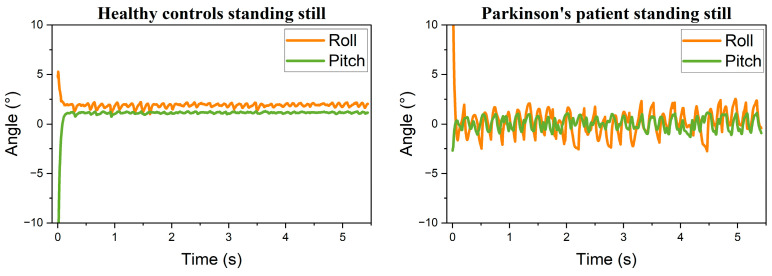
Resting tremor: healthy controls vs. PD patients.

**Figure 6:**
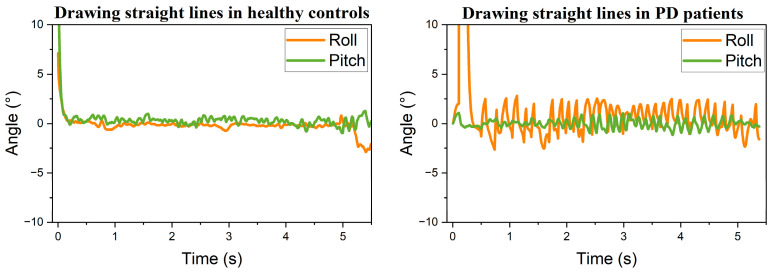
Air-drawn straight lines: healthy controls vs. PD patients.

**Figure 7:**
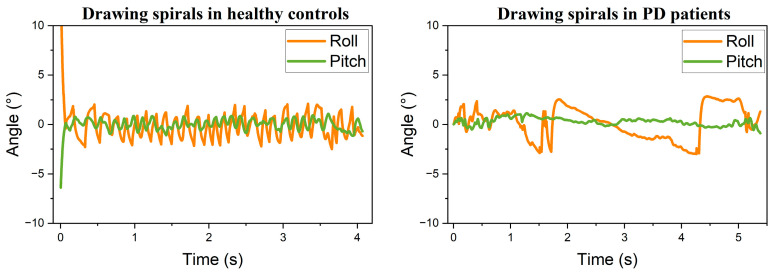
Air-drawn spirals: healthy controls vs. PD patients.

Following the attitude transformation, the processed data were visualized as trend curves, as shown in Figure 5, Figure 6 and Figure 7. Comparison between static conditions and mid-air straight-line drawing reveals that PD patients exhibit larger amplitude variations in both roll and pitch angles compared to healthy participants, with this distinction remaining pronounced during dynamic tasks. These observations indicate that healthy individuals exhibit minimal hand tremor during stationary periods, whereas patients with PD demonstrate more substantial oscillations. During the mid-air spiral drawing task, movement of the sensing device along the Z-axis resulted in detectable fluctuations in roll angle among healthy participants. Nevertheless, the overall variation in postural angles remained consistently smoother in healthy controls compared to patients with PD. In summary, the postural angle curves of healthy individuals demonstrate greater smoothness than those of PD patients across both static and dynamic task conditions.

### 4.5. Data Partitioning

The sensor data constitute a continuous time series. To accommodate sequences exceeding the model’s processing capacity, we segmented the data into manageable intervals for efficient model input. The time-series data, plotted with time on the x-axis, comprises two motion features: roll and pitch angles. Our objective is to compute these values over specified time windows and utilize them for binary classification of PD presence. A sliding window approach was employed for data segmentation. By defining a fixed stride length and advancing the window along the temporal sequence, the data were partitioned into multiple overlapping segments. The mathematical formulation for generating these subsequences is provided as follows.

(15) $$num\_subseqs=\frac{data\_len-1}{num\_steps}$$

Here, num_subseqs represents the number of sub-sequences, data_len denotes the data length, and num_steps indicates the stride. Normal and diseased data are labeled for classification.

### 4.6. Model Training and Evaluation

The dataset was partitioned into training, validation, and test subsets using an 8:1:1 ratio. An LSTM-based architecture was employed as the core model framework. The model accepts preprocessed six-dimensional sensor data as input and generates a two-dimensional probability vector as output, representing the likelihood of the subject belonging to either the healthy control group or the PD group.

**Figure 8:**
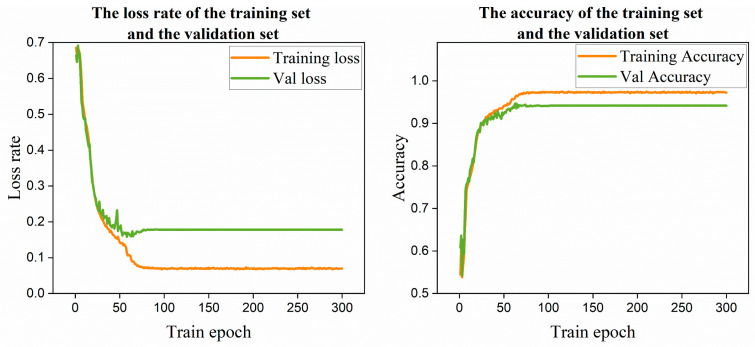
Curves of training loss and validation accuracy.

Throughout the training process, the progression of training loss and validation accuracy across epochs was monitored and recorded, as visualized in Figure 8.

In Figure 8, the training loss decreases rapidly during the initial phase and stabilizes in later epochs, indicating that the model gradually converges as training progresses. The validation loss curve closely aligns with the training loss, though its stabilized value remains slightly higher. It can be observed that both training and validation losses peak around the 50th epoch before stabilizing, and a similar trend is reflected in the accuracy curves.

**Figure 9:**
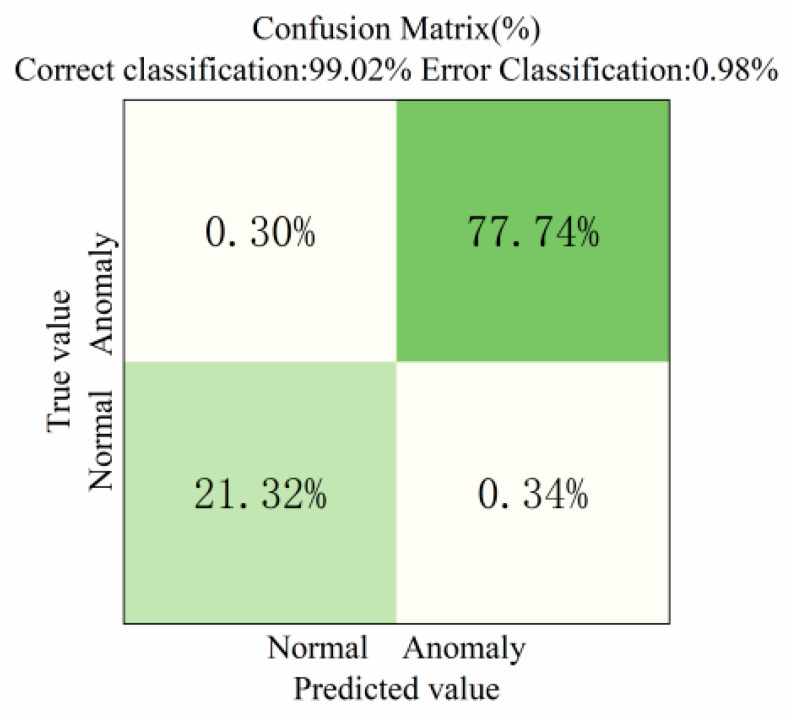
Confusion matrix diagram.

To further evaluate the model’s ability to discriminate between healthy subjects and PD patients based on kinematic tremor data, a confusion matrix of the test set classification results is presented. The matrix displays the frequency of predictions for each actual class across the predicted categories, as shown in Figure 9.

As shown in Figure 9, the model achieved a correct classification rate of 99.02% and an error classification rate of 0.98%, indicating its ability to predict sample categories in most cases accurately. Among healthy individuals, 21.32% of the total data were accurately identified as normal. Among PD patients, 77.74% of the total data were correctly classified. Overall, healthy and diseased individuals were accurately classified, achieving an identification accuracy of 99.06%. However, some samples still exhibited confusion between normal and diseased states. Preliminary analysis suggests this may stem from similar variations in the data collected by the smart wristband.

### 4.7. Classification Performance of LAFNet and Model Variants

To demonstrate the superiority of the proposed LAFNet model, we compared it with four implemented variants: TAFNet, LDLFNet, LDLAFNet, and LDLTFNet. All models share the same LSTM-based backbone but differ in their attention mechanisms and dropout configurations, enabling a comprehensive ablation-style evaluation within our framework.

As shown in Table 2, the five proposed network models are compared against existing state-of-the-art approaches. Among them, the LAFNet model achieves the highest performance, with an accuracy of 99.02%, demonstrating its effectiveness in distinguishing between kinematic tremor data from healthy individuals and those from patients with PD. Furthermore, it was observed that using a linear activation function in the attention mechanism yields better performance than the Tanh activation function. The nonlinearity introduced by Tanh may, in some cases, constrain the expressive power of the attention mechanism. Additionally, applying Dropout after the stacked LSTM layers resulted in noticeably lower accuracy compared to applying Dropout only after the attention mechanism. Although Dropout helps prevent overfitting, excessive use may impair the model’s capacity to retain critical information. Moreover, the combined use of both attention and Dropout layers may lead to instability in learning attention weights, which contributes to the decline in accuracy.

### 4.8. Attention Visualization Results

**Figure 10:**
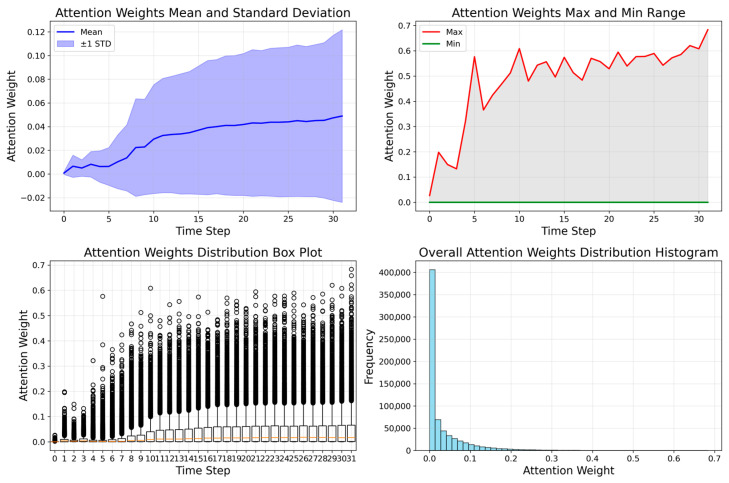
Attention-weight visualizations.

The attention-weight visualizations highlight how LAFNet prioritizes informative temporal segments and channels for binary classification, as shown in Figure 10.

Temporal focus. The attention-weight visualizations indicate a clear temporal preference of LAFNet. Peak weights cluster around steps 29–31 within the 32-step window, implying that the most discriminative cues for separating normal and abnormal patterns reside in the latter half of the sequence.

Weight distribution and stability. The attention histogram exhibits a bimodal shape with most weights in the 0.02–0.05 range, suggesting broadly moderate attention across time with selective amplification of key segments. Box-plot summaries reveal highly consistent attention profiles across samples with few outliers, indicating a robust and stable mechanism over the test cohort. Max–min envelopes further show that, while average attention remains relatively stable, the model adapts its focus intensity, reaching peaks of approximately 0.08–0.10 at the most informative steps.

Clinical interpretation. In the spiral-drawing task, high-weight intervals co-locate with clinically meaningful events: (i) brief, high-frequency tremor bursts (notably at steps 29–31), (ii) irregular rotational transitions in mid-sequence, and (iii) movement initiation/termination phases characterized by lower yet stable attention. This pattern aligns with clinical observations that PD-related motor abnormalities are most pronounced at specific phases of fine-motor execution, particularly during the completion stage.

Technical significance. These visualizations confirm that the attention mechanism captures the temporal dynamics necessary for accurate classification. On the test set comprising 21,441 samples, the overall accuracy reached 98.44%, with the model consistently concentrating on clinically relevant intervals—supporting the effectiveness of the attention-enhanced LSTM architecture for movement-disorder detection.

### 4.9. Diagnosis of PD via Mid-Air Archimedean Curve Drawing

To evaluate the hypothesis that mid-air Archimedean spiral tracing offers superior diagnostic capability compared to conventional paper-based drawing for PD detection, kinematic tremor data were collected from both tasks. The proposed LAFNet model was trained independently on each dataset, with comparative performance results on the test set detailed in Table 3.

As shown in Table 3, the LAFNet model achieved excellent performance in diagnosing PD based on mid-air drawing of Archimedean curves, with an accuracy of 99.02%, precision of 98.71%, recall of 99.35%, and an F1-score of 99.03%. These results indicate that the model effectively captures tremor characteristics associated with PD, demonstrating its strong potential for accurate screening. In contrast, performance declined significantly when the model was applied to paper-based drawing data, with metrics falling to 88.3% accuracy, 84.6% precision, 81.4% recall, and an F1-score of 82.09%. This performance gap may be attributed to the subtlety of early PD symptoms: traces produced by patients in the initial stages exhibit minimal deviation from those of healthy individuals in terms of smoothness, continuity, and consistency, which complicates discrimination by both visual inspection and computational models. Furthermore, Parkinsonian tremor often amplifies under motor fatigue, an effect that may be less pronounced or consistently captured in constrained paper-based tasks.

## 5. Discussion

The experimental results demonstrate that the proposed LAFNet architecture achieves superior performance compared to other methods on the clinical dataset.

### 5.1. Performance Comparison with Existing Models

Existing deep learning approaches typically employ CNNs or standard LSTM architectures on raw accelerometer signals. These models treat all temporal frames equally and lack mechanisms for emphasizing clinically relevant motor events. LAFNet overcomes this limitation through a fused linear-attention module that adaptively highlights informative temporal segments. This design enables substantially more discriminative tremor representation, resulting in superior diagnostic accuracy compared with prior tremor-based PD detection systems.

We compared it with several published deep learning approaches for PD classification based on body sensor data. For instance, Atri et al. (2022) employed a one-dimensional convolutional neural network (1D-CNN) on motion data collected by a Verily Study Watch, achieving approximately 90% accuracy for classifying individual 5-s walking segments [37]. By comparison, the LAFNet model attained an accuracy of 99.02% on the test set, demonstrating not only significantly better performance but also greater practicality and robustness, as it does not require daily data aggregation. Furthermore, LAFNet outperformed the Bi-LSTM with Attention model [38], which achieved an accuracy of 98.00% using accelerometer data. These results indicate that the stacked LSTM architecture combined with an attention mechanism can more effectively capture discriminative features in kinematic tremor data, thereby improving the accuracy of PD diagnosis. It should be noted that other baseline models listed in Table 4, such as the Hybrid Model [39], FB-DNN [40], MLP [41], VGG16 [38], and the voice-based MLP model [39], utilize different data modalities and are not directly comparable. The complete results are presented in Table 4.

### 5.2. Comparison with Traditional Diagnostic Methods and Related Disorders

Compared with traditional diagnostic pathways, our IMU-based approach provides an objective and low-cost means of detecting subtle motor abnormalities that may be missed during routine clinical examination. While DaT/PET imaging remains the gold standard for confirming dopaminergic degeneration, its high cost, radiation exposure, and limited accessibility restrict its use in early screening. The kinematic features extracted by our system also show potential for distinguishing PD from clinically overlapping conditions such as essential tremor (ET), multiple system atrophy (MSA),progressive supranuclear palsy (PSP), and dementia with Lewy bodies (DLB), all of which share early motor manifestations that complicate differential diagnosis. Nevertheless, broader multi-center validation and direct comparison with conventional diagnostic tests are needed to fully establish its clinical applicability.

### 5.3. Limitations and Future Directions

Despite these achievements, certain limitations remain. First, the data acquisition equipment did not utilize magnetometers, resulting in the absence of yaw data, which may affect the model’s ability to represent complex motion trajectories accurately. Second, the collected dataset is relatively limited in size, with insufficient sample coverage to comprehensively describe the diverse tremor patterns observed in Parkinson’s disease patients, which may restrict the model’s generalization capabilities. Furthermore, the current experiment primarily relies on acceleration and angular velocity data, failing to integrate multimodal information (such as electromyography signals or voice data), which may overlook richer diagnostic features.

For future development, we could consider incorporating a magnetometer to obtain precise yaw values. This could be combined with nine-axis sensors—such as accelerometers, gyroscopes, and magnetometers—to enhance motion trajectory reconstruction capabilities. Simultaneously, we could expand the dataset scale to strengthen model robustness. We could also explore multimodal data fusion or transfer learning techniques by integrating (i) ankle-mounted IMU data to capture lower-limb kinematics, (ii) voice recordings for acoustic biomarkers, and (iii) tablet-captured spiral trajectory images/pen kinematics. This approach will adapt to diverse sensors and patient populations, further improving LAFNet’s diagnostic performance.

### 5.4. Clinical Relevance and Impact

This study proposes a method for early detection of PD by acquiring mid-air hand tremor data using a wrist-worn device and applying the LAFNet model. Mid-air spiral tracing achieves 99.02% diagnostic accuracy, providing critical advantages for clinical deployment.

#### 5.4.1. Early Intervention Window Enhancement

By the time a patient is first clinically diagnosed with PD, a significant proportion (50–70%) of the cells in the substantia nigra have already been lost [45]. This study proposes an air-based tracing of an Archimedean spiral instead of on paper drawing. The absence of a fixed support point during mid-air drawing increases hand fatigue and tremor susceptibility, thereby enhancing the sensitivity for detecting subtle motor impairments in the early stages of PD (Hoehn & Yahr stages 1–2). Our method allows for identifying PD during its prodromal phase, providing an extended diagnostic window for timely and effective intervention. This temporal advantage holds substantial clinical importance, as early initiation of neuroprotective interventions such as dopamine agonists and monoamine oxidase-B inhibitors during the initial stages of neurodegenerative diseases has been shown to be significantly more effective than delayed treatment [46].

#### 5.4.2. Objective Quantification for Clinical Decision-Making

Currently, clinical evaluation of PD primarily relies on the Unified Parkinson’s Disease Rating Scale (UPDRS) and the Hoehn and Yahr (H&Y) scale. However, due to their time-consuming, subjective, and infrequent nature, as well as their dependence on patients’ recall, these assessments are not routinely employed in clinical practice [47]. Our system provides standardized and reproducible measurements, offering an objective assessment that enables more precise monitoring of disease progression compared with traditional clinical rating scales, and facilitates optimized medication titration based on quantitative tremor metrics.

#### 5.4.3. Enhancing Accessibility and Screening

The portability and affordability of the smart wristband render the proposed system highly suitable for deployment in primary care settings, community health centers, and even home-based assessments. Such accessibility could substantially enhance large-scale screening, particularly in underserved regions with limited access to Parkinson’s disease specialists. From a health economics perspective, early detection of PD translates into considerable cost savings, as timely diagnosis and intervention can significantly reduce lifetime healthcare expenditures.

#### 5.4.4. Patient-Centered Outcomes and Quality of Life

The proposed method has the potential to shorten the diagnostic timeline of Parkinson’s disease. Initiating exercise interventions during the early stages of PD may help delay disability progression and enhance patients’ quality of life.

## 6. Conclusions

This paper presents a novel approach for the intelligent diagnosis of early-stage PD based on kinematic data acquired during mid-air drawing of Archimedean spirals. To support this task, we designed LAFNet, an LSTM network integrated with an attention mechanism, which effectively captures tremor-related patterns and motor control abnormalities exhibited by PD patients during the drawing process. The experimental results show that the proposed method achieves high diagnostic performance, obtaining an accuracy of 99.02%, precision of 98.71%, recall of 99.35%, and an F1-score of 99.03% on the test set. These results significantly outperform multiple state-of-the-art models and several hybrid architectures, indicating the model’s strong capacity for feature extraction and classification. The study offers a novel and effective computer-aided diagnostic solution for early screening of PD, demonstrating considerable potential in terms of both accuracy and clinical applicability.

## Figures and Tables

**Table 1:** Baseline characteristics of the study population.

Characteristics	Hoehn & Yahr Stage	Age	Sex (M/F)	PD Duration (Years)	Side of Symptom Onset	Main Initial Motor Symptom	N
Early PD	1 1 1 1 1.5 2	45 ± 5 55 ± 5 60 ± 4.2 67 ± 5.1 65 ± 3.1 70 ± 4.6	59.45% / 40.55%	3.2 ± 1.5	Right 22 (44%) Left 18 (36%) Bilateral 10 (20%)	Tremor-dominant 21 (42%) Bradykinesia-dominant 17 (34%) Rigidity-dominant 12 (24%)	2 3 7 15 13 10
Healthy Controls	-	50 ± 5	56%/44%	-	-	-	25
	-	60 ± 5	54%/46%	-	-	-	26

**Table 2:** Evaluation results of different models for PD diagnosis.

Model	Description	F1-Score	Recall	Precision	Accuracy
LAFNet	LSTM + Attention	99.03	99.35	98.71	99.02
TAFNet	Transformer	98.25	98.15	98.34	98.23
LDLFNet	Low-dim bottleneck + Late fusion	97.48	97.43	97.53	97.47
LDLAFNet	Attentive low-dim late fusion	97.2	97.43	96.97	97.25
LDLTFNet	Compact late fusion + Lightweight	96.78	96.82	96.73	96.88

**Table 3:** Performance comparison of Archimedean spiral drawing on paper and mid-air.

Methods	Accuracy	Precision	Recall	F1-Score
Drawing in the air	99.02%	98.71%	99.35%	99.03%
Drawing on paper	88.30%	84.60%	81.40%	82.09%

**Table 4:** Evaluation results of different published models for PD diagnosis.

Model	Data Type	F1-Score	Recall	Precision	Accuracy
1D-CNN [39]	Motion	–	–	–	90
Bi-LSTM with Attention [40]	Motion	98	98	99	98
	Voice	91.13	92.5	89.84	91.11
FB-DNN [42]	Voice	98	98	98	96.15
MLP [43]	Voice	99	98	100	98.31
VGG16 [13]	Voice	–	–	–	91.8
MLP [44]	Voice	78.5	67.6	93.4	71.5

Note: The data used by the five models in this article are all kinematic tremor data. Some of the models in the table are implemented based on speech data, and there are certain differences in the data modalities.

## Data Availability

The data that support the findings of this study are available in the main text and the figures. Source data are provided within this paper. The datasets generated and/or analyzed during the current study are available from the corresponding authors on request. Our code, data, and model checkpoints are available here: https://github.com/cropsoft-shi/Parkinson (accessed on 14 October 2025).
